# The Anatomical and Functional Outcome of Autologous Internal Limiting Membrane Transplant With 27-Gauge Vitrectomy in Failed and Recurrent Macular Holes in a Spectrum of Pathologies

**DOI:** 10.7759/cureus.76120

**Published:** 2024-12-21

**Authors:** Fatima Mohsin, Muhammad Amer Awan

**Affiliations:** 1 Ophthalmology, Shifa International Hospitals Limited, Islamabad, PAK; 2 Ophthalmology, Shifa College of Medicine, Shifa Tameer-e-Millat University, Islamabad, PAK

**Keywords:** 27-gauge vitrectomy system, autologous ilm transplant, failed macular hole, recurrent macular hole, refractory macular hole, vitreoretinal surgery

## Abstract

Objective: To describe the anatomical and functional outcome of autologous internal limiting membrane (ILM) transplant with 27-gauge plus (27G+) three ports pars plana vitrectomy (PPV) in failed and recurrent full-thickness macular holes (MH) in a spectrum of pathologies.

Study design: Observational cohort study

Methods: Seven eyes of seven patients who had failed or recurrent MH were included from January 2017 to January 2022. A single vitreoretinal surgeon performed all surgeries using a 27G+ PPV system in a tertiary care hospital. An autologous ILM transplant was performed in each case, using hexafluoroethane or perfluoropropane as tamponade agents. The primary outcomes encompassed achieving MH closure, determining the best possible vision achieved during the 12-month follow-up period, and assessing any post-operative complications.

Results: The average age of the patients enrolled in this study was 49.4 years. Four of the cohort of seven patients were male, while three were female. All individuals underwent successful surgery, with the closure of MH postoperatively. Swept-source optical coherence tomography was used to examine the closure of the MH on the first day after surgery, which was further validated by the absorption of gas. Over 12 months, the mean best corrected visual acuity in the logarithm of the minimum angle of resolution (Log MAR) improved significantly, progressing from 1.60 to 0.60. This favorable trend persisted throughout the 12-month follow-up, with MH remaining consistently closed. One patient encountered a subsequent retinal re-detachment, necessitating prompt and effective surgical intervention, resulting in successful management.

Conclusion: An autologous ILM transplant with a 27G+ PPV is a delicate and effective technique for closing MH in complicated cases. We recommend this technique for failed and recurrent MH in different pathologies.

## Introduction

In 1991, Kelly and Wendel were the first to introduce three ports pars plana vitrectomy (PPV), the removal of posterior hyaloid along with epiretinal membranes, and gas tamponade as a surgical treatment for full-thickness MH [1]. Since then, many surgeons have also described the role of the internal limiting membrane (ILM) peeling in the success of MH surgery. Therefore, PPV and ILM peeling has now become a gold standard, with more than 90 % closure of MH [2]. Chronic, large MH, concurrent retinal detachment, myopia, incomplete vitrectomy, short duration of gas tamponade, and failure to comply with post-procedure positioning are some of the risk factors that are responsible for refractory MH that remain open or fail to close after primary surgery [2,3].

Closure of persistent and recurrent MH is challenging, and over time, surgeons have explored various techniques to close these holes. These include light and heavy silicon oils, whole blood derivatives, autologous retinal transplant, anterior capsular flaps, ILM, and human amniotic membrane (hAM) grafts have also been proposed [4-6].

Morizane et al. performed successful ILM transplantation in 10 eyes [7]. The ILM grafts are believed to function as a scaffold that speeds up the process of muller cell gliosis, thereby closing the macular hole [8]. Our study aims to state the anatomical and functional outcomes of autologous ILM transplant with 27-gauge plus (27G+) PPV in patients with refractory or recurrent MH in a spectrum of pathologies.

## Materials and methods

This is an observational cohort study of individuals with chronic, refractory, and recurrent MH treated using 27G+ PPV, autologous ILM transplant, and tamponade agent hexafluoroethane (C2F6) or octafluoropropane (C3F8). With the approval of the Institutional Review Board (IRB) and Ethics Committee, data from seven patients' medical records were retrieved. The best corrected visual acuity (BCVA) before and 12 months after the surgery was recorded. All of them had a detailed eye exam by the same vitreoretinal specialist, and swept-source optical coherence tomography (SS-OCT) scans were taken before surgery, on the first post-op day, one week, four weeks, three months, and six months after the ILM transplant. The base diameter of these full-thickness MH was measured. Written informed consent was taken before surgery. Age, gender, laterality, the type of refractory MH, and post-operative complications were also obtained. The date and duration of the operation, kind of anesthetic, surgical steps, use of laser/cryotherapy, and type of tamponade agent used were all noted. Posture recommendations provided to the patient shortly following surgery were documented.

Surgical technique

Patients were administered a modified retrobulbar block (25G into a 1-inch needle was used to inject through fornix inferotemporally) and given a combination of 1% lignocaine and 0.5% bupivacaine or general anesthesia chosen based on individual needs. Supplementary bupivacaine 0.5% was introduced through sub-tenon injections as necessary. The surgical procedure employed the 27G+ PPV constellation system from Alcon Laboratories (Fort Worth, TX) for all patients. The non-contact wide-angle viewing system BIOM from Oculus Inc. (Wetzlar, Germany) was utilized for optimal visualization of the fundus. Notably, instances of visually significant cataracts necessitated combined cataract extraction via phacoemulsification and vitrectomy. A relatively identical surgical strategy was used in all of the eyes. However, depending on the etiology and difficulties observed, slightly different techniques were performed in some eyes. A single VR surgeon performed all of the procedures. All eyes were previously vitrectomized.

In each instance, the ILM was subjected to a two-minute staining process using ILM-BLUE® dye, developed by the Dutch Ophthalmic Research Center (DORC) located in Zuidland, Netherlands. Keeping the intraocular pressure of 20 mmHg, after enlargement of ILM peel, a piece of ILM tissue about 2-disc areas in size, distant from the macular hole, was peeled and carefully placed within the hole using Eckardt ILM forceps. In two eyes, perfluorocarbon liquid (PFCL) was also used to stabilize the graft over the MH. Care was taken not to disturb the underlying retinal pigment epithelium in the base of the hole during graft placement and manipulation. Additional procedures such as cryotherapy and endo laser were performed in one case each. A fluid air or PFCL air exchange was then carried out, followed by using intraocular gas, either C2F6 or C3F8 as a tamponade agent. The choice of gas was made according to the size of refractory MH (greater than or equal to 900 µm).

At the end of the surgery, subconjunctival antibiotics (gentamycin) and corticosteroids (dexamethasone) were given. Topical steroids, antibiotics, and cycloplegic eye drops were advised postoperatively. All patients were counseled to maintain a face-down position for an average of 14-16 hours after surgery.

After the surgical procedure, a comprehensive ocular examination was conducted for each patient. These evaluations occurred on the first postoperative day and subsequently at one week, one month, three months, six months, and 12 months after surgery. The eyes with partial or no closure, as determined by an OCT scan on day one, were recommended to maintain a face-down posture for one week. Following complete gas absorption, BCVA was evaluated using the logarithm of the minimum angle of resolution (Log MAR) at three, six, and 12 months. To determine MH closure, OCT macula was carried out on post-op day one and again following gas resorption. All patients were followed for 12 months. The primary outcome of this paper was complete MH closure, which was determined on SS-OCT scans postoperatively and BCVA.

Statistical analysis

All collected data underwent rigorous recording and subsequent analysis utilizing the Statistical Product and Service Solutions (SPSS, version 21.0; IBM SPSS Statistics for Windows, Armonk, NY) software. Qualitative datasets were meticulously scrutinized for percentages and frequencies, whereas quantitative variables were characterized by mean and standard deviation. Counting finger vision was changed to 1.98 in Log MAR [9].

## Results

The study encompassed a cohort of seven patients whose comprehensive information has been given in Table 1. The average age of the participants, comprising four males and three females, was 49.4 years, with an age range spanning from 14 to 73 years. Before the transplantation of the ILM, the mean base diameter of the MH was measured to be 1092 ± 338 µm, with a range of 817-1,721 µm.

**Table 1:** Types of refractory macular holes, tamponade, and outcome Demographic and clinical characteristics of patients with refractory MH undergoing autologous ILM transplantation MH = macular hole, µm = microns, RD = retinal detachment, ILM = internal limiting membrane, C3F8 = octafluoropropane, C2F6 = hexaflouroethane

Type of refractory MH	Base diameter of MH (microns)	Tamponade used	Face-down posturing (time in hours)	MH status at 12 months follow-up
Idiopathic	900	14% C3F8 gas	14	Closed
Idiopathic	821	18%C2F6 gas	18	Closed
Idiopathic	817	18% C2F6 gas	10	Closed
Idiopathic	1387	16% C3F8 gas	24	Closed
RD associated	975	16% C3F8 gas	12	Closed
RD associated	1026	14% C3F8 gas	12	Closed
Traumatic	1721	16% C3F8 gas	10	Closed

The average BCVA before the operation was 1.60. Only one patient received general anesthesia, while the rest of the procedures were performed under the modified retrobulbar block. No anesthesia-related complications were observed. All eyes had previous PPV and ILM peel. Two eyes had combined phacoemulsification cataract extraction with intraocular lens implantation and autologous ILM transplant. The remaining eyes were pseudophakic.

At the end of the surgical procedure, C2F6 gas in 28% (n=2) and C3F8 gas in 72% (n=5) of the patients were used as tamponade agents. The mean face-down posturing time advised was 14 ± 5 hours after surgery.

A patient with previous retinal detachment surgery developed retinal re-detachment within a month of autologous ILM transplantation, but MH remained closed. However, it was successfully managed by another surgery, and silicon oil was used as a tamponade agent.

**Figure 1:**
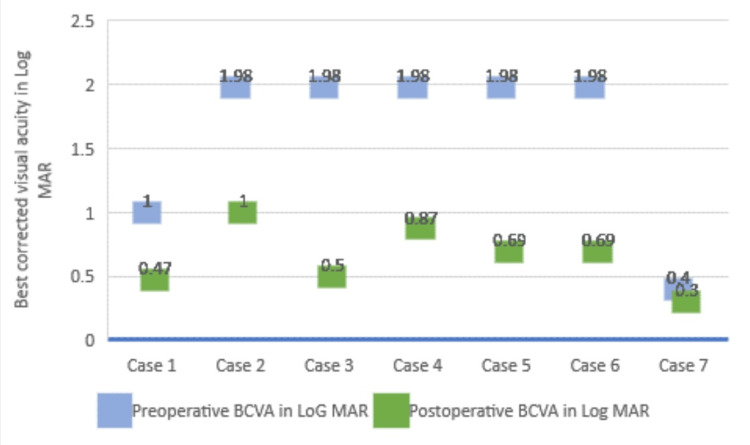
Preoperative and postoperative visual acuity after ILM transplant This box plot graph compares preoperative and postoperative best-corrected visual acuities (BCVAs) in Log MAR of 07 eyes with refractory macular holes that underwent autologous transplantation of the internal limiting membrane.

**Figure 2:**
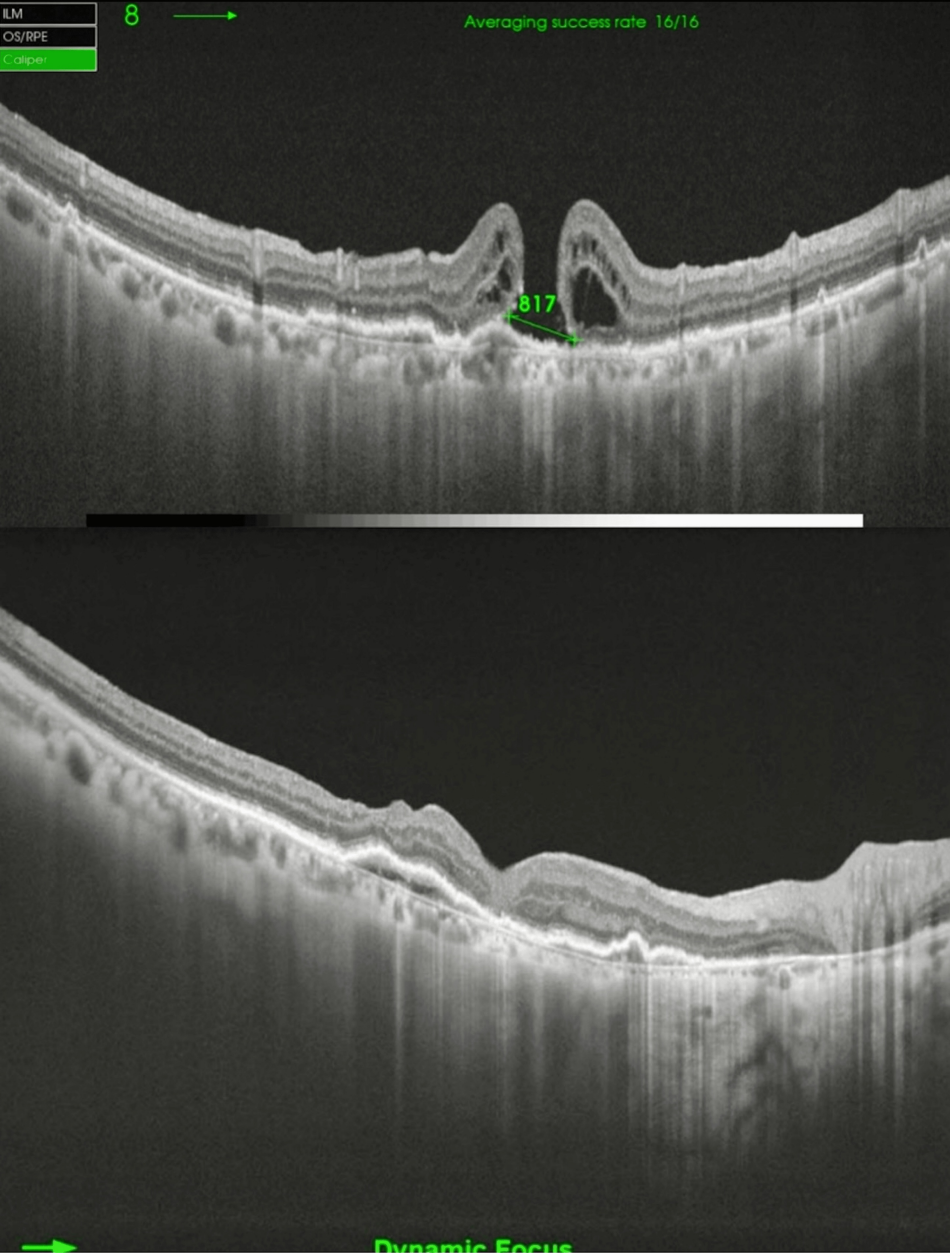
Outcome of refractory macular hole after failed idiopathic macular hole surgery Preop and postop OCT macula of the patient who underwent MH surgery. The base diameter of the MH was 817 µm. MH = macular hole

**Figure 3:**
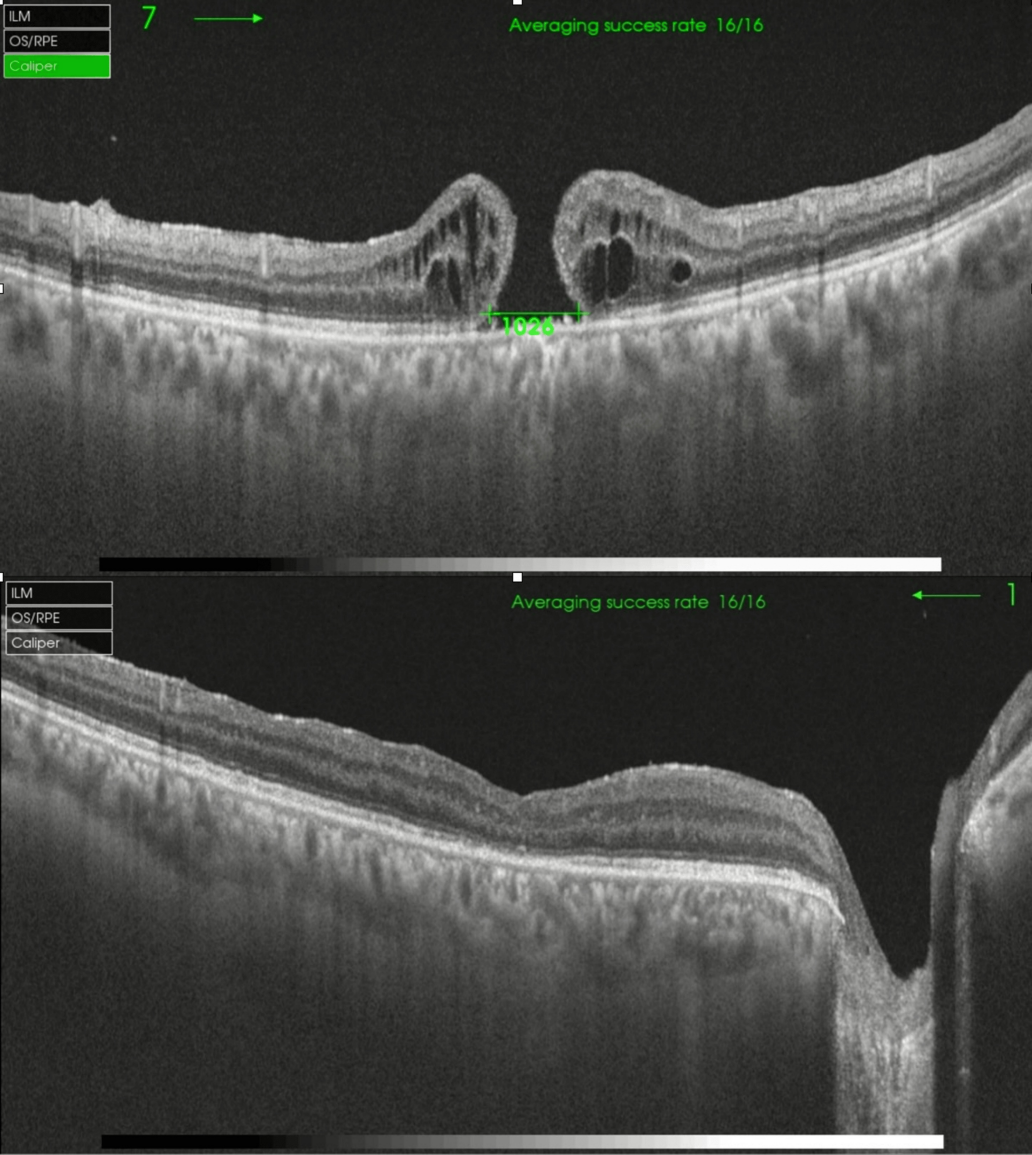
Outcome of the refractory macular hole after failed post-retinal detachment surgery Preop and postop OCT macula of the second patient who developed MH after retinal detachment surgery and was successfully treated with an ILM transplant. The base diameter of the MH was 1026 µm. MH = macular hole, ILM = internal limiting membrane

**Figure 4:**
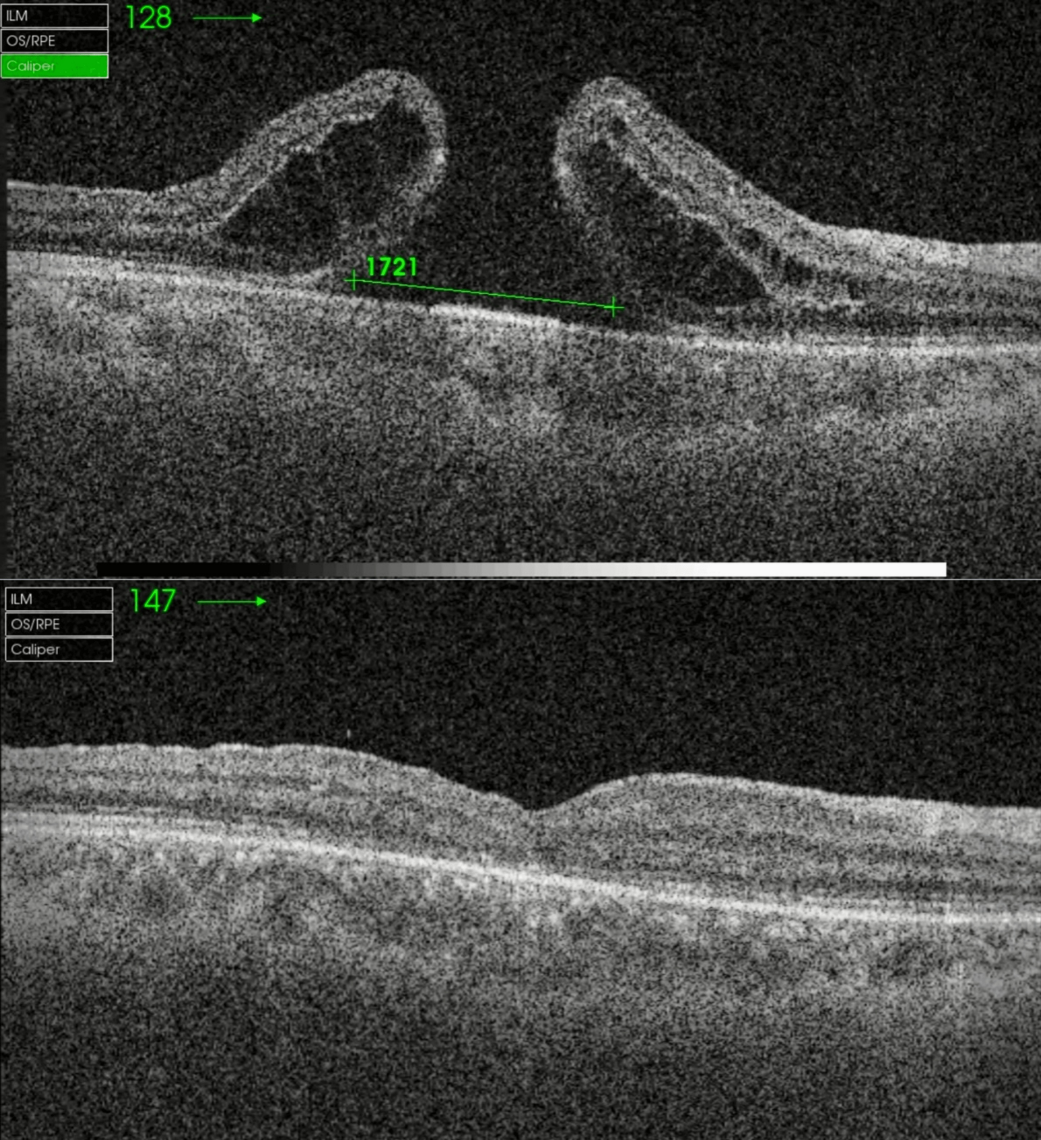
Outcome of the refractory macular hole after failed traumatic macular hole surgery Preop and postop OCT macula of the patient who underwent traumatic MH surgery. The base diameter of the hole is the largest in this study, namely, 1,721 µm.

Twelve months post ILM transplantation, the MH remained closed and BCVA improved from 1.98 to 1. At six months, the mean BCVA had significantly improved to 0.60 from baseline and maintained the same level at 12 months (Figure 1). Anatomically, MH remained closed when evaluated at three to 12 months after surgery on OCT scans (Figures 2-4).

## Discussion

Refractory MH has an incidence of about 4-11% after the previous PPV with or without ILM peeling and is associated with lower success rates of closure [10]. If untreated, the visual acuity of these patients may worsen eventually. The challenge arises when the ILM gets peeled off in the previous retinal surgery, and the patient presents with a refractory or recurrent large and chronic MH having a base diameter of more than 400 µm. Surgeons, with time, have employed various methods to improve the visual outcome in such cases. Anterior lens capsule, autologous retinal transplantation, ILM transplantation, autologous blood product, and amniotic membrane flap have all been used as a scaffold with various success rates and complications [4,5,11].

In the literature, work on ILM transplantation has been done on refractory MH. Morizane conducted a preliminary study involving 10 patients afflicted with large, chronic macular holes due to trauma, proliferative diabetic retinopathy, and macular foveoschisis. He pioneered the technique of autologous ILM transplantation and accomplished a notable success of 90% [12]. We conducted this study focusing on the patients with large, refractory, or reopened MH (greater than 800µm) in idiopathic, associated with RD and traumatic, and achieved a 100 % closure rate. Previous PPV and ILM peeling had been unsuccessful in closing these MH initially.

ILM transplantation has been described previously as an adjunctive surgical technique in addition to PPV and ILM peeling for large, chronic idiopathic macular holes [2]. De Novelli et al. conducted a study in 2015 that showed successful closure of large, chronic, idiopathic, and refractory macular holes in 10 eyes. Their study included idiopathic primary MH and refractory MH [13]. However, our study contributed to the fact that ILM tissue can be used successfully to treat different types of refractory or reopened MH, such as idiopathic, traumatic, and secondary MH associated with retinal detachment.

Various techniques have been compared previously to close these recalcitrant MH. One such study compared the use of hAM, autologous ILM, and retinal tissue as a treatment for refractory MH. They reported a 90% success rate in the closure of MH with autologous ILM transplantation and achieved a mean BCVA of 0.69 [3]. Lorenzi et al. showed a higher closure rate with hAM graft than with autologous ILM transplants [3]. However, in this study, we achieved a 100% closure rate where the mean BCVA also improved to 0.60. We believe the ILM graft acted as a scaffold for the cells to proliferate and close MH.

As reported in the literature, surgeons used 23 and 25-gauge PPV systems for autologous ILM transplantation surgery [3,14]. Our study employed 27G+ PPV, one of the most recent, advanced, and efficient smallest gauge vitrectomy systems available. The efficacy of this system in MH surgeries has been demonstrated in the past [15]. Our study is the only one that showed good surgical and visual outcomes after ILM grafting using a 27G+ PPV system.

Dai et al. showed successful ILM transplantation in 13 eyes with large, unclosed macular holes. They utilized a 23-gauge vitrectomy system, followed by indocyanine-green dye (ICG) to stain the ILM and then C3F8 gas as tamponade [14]. Our study demonstrated successful MH closure using a 27-gauge vitrectomy system where ILM-BLUE® dye was employed for tissue staining that does not cause retinal toxicity compared to ICG [16]. Our study also showed that C2F6 gas is a reasonable option in MH smaller than 900 µm and C3F8 gas is greater than and equal to 900 µm. Our paper emphasizes the need to re-operate on patients with recalcitrant large macular holes using autologous ILM tissue.

## Conclusions

Despite the limited sample size and retrospective nature, it has provided strong evidence supporting the ILM tissue graft as a safe and effective option for closing large refractory and reopened MH in various pathologies. A 27G+ PPV system has been proven useful even in these complex cases.

## References

[1] Morales-Canton V, Meizner-Grezemkovsky D, Baquero-Ospina P, Crim N, Wu L (2023). Optical coherence tomography angiography in macular holes autologous retinal transplant. J Clin Med.

[2] Ma FY, Xi RJ, Chen PF, Hao YH (2019). Free autologous internal limiting membrane transplantation in the treatment of large macular hole. Int J Ophthalmol.

[3] Lorenzi U, Mehech J, Caporossi T (2022). A retrospective, multicenter study on the management of macular holes without residual internal limiting membrane: the refractory macular hole (ReMaHo) study. Graefes Arch Clin Exp Ophthalmol.

[4] Romano MR, Rossi T, Borgia A, Catania F, Sorrentino T, Ferrara M (2022). Management of refractory and recurrent macular holes: a comprehensive review. Surv Ophthalmol.

[5] Cisiecki S, Bonińska K, Bednarski M (2021). Autologous lens capsule flap transplantation for persistent macular holes. J Ophthalmol.

[6] Yamada K, Maeno T, Kusaka S, Arroyo JG, Yamada M (2020). Recalcitrant macular hole closure by autologous retinal transplant using the peripheral retina. Clin Ophthalmol.

[7] Morizane Y, Shiraga F, Kimura S (2014). Autologous transplantation of the internal limiting membrane for refractory macular holes. Am J Ophthalmol.

[8] Giansanti F, Tartaro R, Caporossi T, Bacherini D, Savastano A, Barca F, Rizzo S (2019). An internal limiting membrane plug and gas endotamponade for recurrent or persistent macular hole. J Ophthalmol.

[9] Lange C, Feltgen N, Junker B, Schulze-Bonsel K, Bach M (2009). Resolving the clinical acuity categories “hand motion” and “counting fingers” using the Freiburg visual acuity test (FrACT). Graefes Arch Clin Exp Ophthalmol.

[10] Michalewska Z, Michalewski J, Adelman RA, Nawrocki J (2010). Inverted internal limiting membrane flap technique for large macular holes. Ophthalmology.

[11] Dhami A, Biswas RK, Dogra M, Singh R, Mittal S, Ratra D (2022). Comparison of three techniques of harvesting full-thickness retinal tissue for large or persistent macular holes. Indian J Ophthalmol.

[12] Alezzandrini A, Dorrego CI, Cibrán MV, Cortina-Revelli V, Rocco FD, Zas M, Wu L (2021). A 24 month follow-up of refractory macular holes treated with an autologous transplantation of internal limiting membrane versus retina expansion technique. Int J Retina Vitreous.

[13] De Novelli FJ, Preti RC, Ribeiro Monteiro ML, Pelayes DE, Junqueira Nóbrega M, Takahashi WY (2015). Autologous internal limiting membrane fragment transplantation for large, chronic, and refractory macular holes. Ophthalmic Res.

[14] Dai Y, Dong F, Zhang X, Yang Z (2016). Internal limiting membrane transplantation for unclosed and large macular holes. Graefes Arch Clin Exp Ophthalmol.

[15] Awan MA, Shaheen F, Haq A, Fatima S (2022). The clinical and safety outcomes of 27 gauge pars plana vitrectomy in eyes with macular hole. Cureus.

[16] Li SS, You R, Li M, Guo XX, Zhao L, Wang YL, Chen X (2019). Internal limiting membrane peeling with different dyes in the surgery of idiopathic macular hole: a systematic review of literature and network meta-analysis. Int J Ophthalmol.

